# Humidity and temperature interact in breaking physical dormancy in Fabaceae seeds from the Brazilian semiarid region

**DOI:** 10.1002/ajb2.70254

**Published:** 2026-09-04

**Authors:** Humberto Araújo Almeida, Jessyca Adelle Silva Santos, Queila Souza Garcia

**Affiliations:** ^1^ Laboratório de Fisiologia Vegetal, Departamento de Botânica Universidade Federal de Minas Gerais Belo Horizonte 31270‐901 Minas Gerais Brazil; ^2^ Centro Nacional de Conservação da Flora Instituto de Pesquisas Jardim Botânico do Rio de Janeiro Rio de Janeiro 22460‐030 Brazil

**Keywords:** Caatinga, climatic seasonality, legumes, physical dormancy, seed bank

## Abstract

**Premise:**

In semiarid ecosystems such as the Brazilian Caatinga, physical seed dormancy (PY) is critical for plant persistence when water is unpredictable, but how soil moisture and temperature regulate PY release and seed longevity in species of native Fabaceae that have different growth forms is unknown.

**Methods:**

Seeds of the herbaceous species *Centrosema pascuorum* and *Crotalaria retusa* and tree species *Mimosa ophthalmocentra*, *M. tenuiflora*, and *Piptadenia retusa* were collected in the Caatinga and tested in the field and laboratory to assess dormancy, viability, and germination. Seeds were either buried at the collection site and periodically exhumed over 15 months, or stored dry or with wet–dry cycles at constant or fluctuating temperatures.

**Results:**

Seeds of herbaceous species had rapid PY release and seed depletion shortly after the onset of rainfall, leading to synchronized seedling emergence. Seeds of the trees *M. ophthalmocentra* and *M. tenuiflora* maintained high dormancy and viability, indicating strong potential for persistent soil seed banks, whereas seeds of *Piptadenia retusa* had low persistence due to rapid dormancy loss and high mortality. Across species, PY release was maximized under wet–dry cycles at high temperatures, revealing a synergistic effect of moisture pulses and heat.

**Conclusions:**

Temperature–moisture interactions characteristic of the Caatinga control PY release and seed persistence, with growth form shaping contrasting regeneration strategies. The delayed release of PY in trees spreads germination over time, enhancing persistence under unpredictable rainfall. Early PY release in herbs optimizes exploitation of short favorable windows to complete their rapid life cycle.

Seed dormancy is an adaptive strategy that enhances long‐term plant survival by delaying germination until environmental conditions become favorable (Baskin and Baskin, 2014; Jayasuriya et al., 2015). In semiarid ecosystems with irregular rainfall, such as seasonally dry tropical forests (SDTF), dormancy plays a crucial role in spreading germination over time, synchronizing it with intermittent windows of moisture availability (van Klinken and Goulier, 2013; Just et al., 2024).

Seeds of different species have different types of dormancy, categorized based on physiological and anatomical criteria into five main classes: physiological, morphological, morphophysiological, physical, and combinational (physical + physiological; Baskin and Baskin, 2004). Among these types, physical dormancy (PY) stands out as particularly common across a wide range of species inhabiting semiarid ecosystems (van Klinken and Goulier, 2013; Baskin and Baskin, 2014; Collette and Ooi, 2021). Seeds with PY are characterized by a water‐impermeable seed coat that prevents imbibition, thereby inhibiting germination even when environmental conditions are otherwise favorable (Baskin et al., 2000).

Seeds with PY have specialized morphoanatomical structures known as water gaps—weak zones in the seed coat that respond to environmental cues by undergoing physical or chemical changes. These changes eventually allow water uptake, thereby initiating germination (Baskin et al., 2000; Gama‐Arachchige et al., 2013). However, until environmental conditions become suitable for the opening of water gaps, seeds with PY may remain dormant in the soil for extended periods (Zalamea et al., 2018; Zhang et al., 2020; Hubert et al., 2024). In this context, the presence of PY has been frequently associated with the long‐term maintenance of seed viability within the soil seed bank (Jaganathan, 2016; Miller et al., 2019; Huss and Gierlinger, 2021; Hubert et al., 2024; Jaganathan and Harrison, 2024; Santos et al., 2024). Physical dormancy is widespread across several plant lineages and is especially prevalent in the Fabaceae family (Baskin et al., 2000), a taxonomically and ecologically diverse group highly represented in tropical semiarid environments (Queiroz et al., 2017). Studies on Fabaceae seeds have demonstrated that variations in temperature and soil moisture can induce structural changes in the seed coat, triggering the opening of water gaps and thereby overcoming PY (Matheus et al., 2017; Rodrigues‐Junior et al., 2018; Zhang et al., 2020).

The largest tropical dry ecoregion in South America contains an important area of the SDTF domain, known as the Caatinga (Moro et al., 2016). This region is characterized by an irregular rainfall regime, low annual precipitation, high temperatures, and elevated evapotranspiration rates (Moro et al., 2016; Queiroz et al., 2017; Dexter et al., 2018). Fabaceae is the most abundant and diverse plant family in the Caatinga, comprising both tree and herbaceous species distributed across a range of microhabitats (Queiroz et al., 2017; Morim et al., 2024). Many Fabaceae species in the Caatinga produce seeds with PY, meaning that a substantial proportion of seeds are dispersed in a dormant state (Meiado, 2014). In this context, the regeneration of much of the Caatinga plant biodiversity through the soil seed bank depends on specific environmental conditions that promote PY break (Souza et al., 2020).

In SDTF such as the Caatinga, the formation of soil seed banks is a key factor in maintaining plant biodiversity (Meiado, 2014). The temporal spread of germination increases the likelihood of seedling establishment during sporadic rainfall events, thereby enhancing survival in highly unpredictable environments (Meiado, 2014; Kiss et al., 2018; Moro et al., 2024). However, species with different growth forms may exhibit distinct germination strategies and responses to environmental cues (Donohue et al., 2010; Vandelook et al., 2012; Kadereit et al., 2017). In general, due to their short life cycle, annual herbs from semiarid environments tend to germinate more rapidly than tree species, maximizing their ability to complete their life cycle within the brief and irregular periods of soil moisture availability (Scott and Morgan, 2012; Compagnoni et al., 2021). Accordingly, seeds of herbaceous species dispersed with an impermeable seed coat are expected to lose dormancy more rapidly than those of tree species.

Understanding the dynamics of dormancy release and in situ seed longevity is therefore essential for elucidating vegetation recruitment processes in seasonally dry ecosystems. Despite this relevance, significant knowledge gaps remain regarding how environmental conditions influence the release of primary dormancy and seed persistence in Caatinga species. In particular, little is known about potential differences in the dynamics of physical dormancy (PY) release between herbaceous and tree species, which often have contrasting life‐history strategies and recruitment patterns in semiarid environments.

To address this gap, we collected seeds from two herbaceous and three tree species of Fabaceae that exhibit PY in the Caatinga to investigate how environmental factors influence dormancy release over time and assess their potential to form a soil seed bank. We used the seeds in a series of laboratory and field experiments designed to address the following questions: How does the irregular water availability of the Caatinga affect the release of PY and in situ seed longevity in Fabaceae? What roles do temperature and wet–dry cycles play in overcoming PY in Fabaceae seeds from the Caatinga? We hypothesized that PY contributes to seed longevity in the soil and that interactions between temporal variation in temperature and moisture—typical in the Caatinga—promote a gradual release of dormancy, resulting in temporally dispersed germination that varies among species according to their growth form.

## MATERIALS AND METHODS

### Studied Fabaceae species

**Figure 1:**
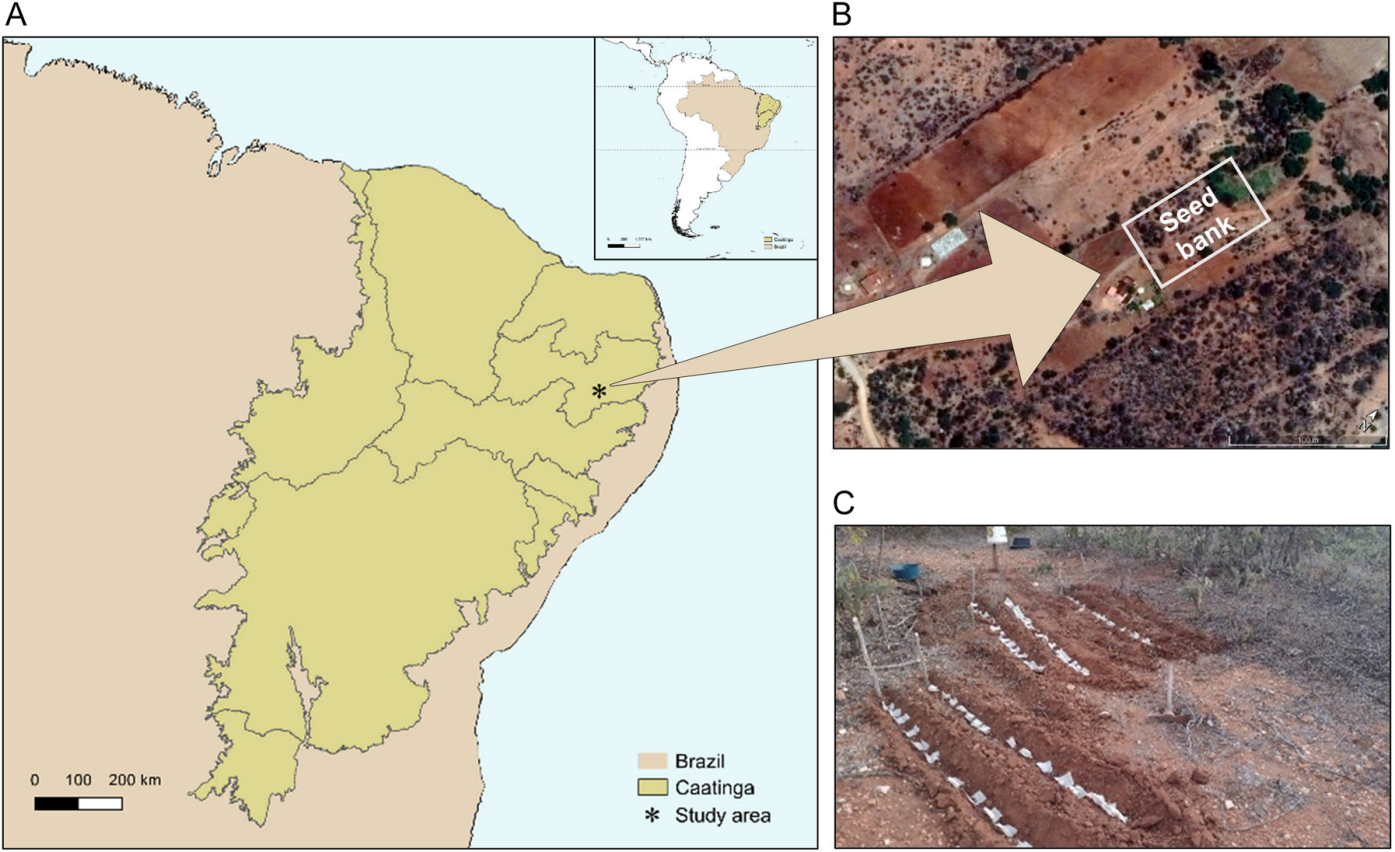
Location of the experimental site (7°23'36.82“S; 36°26'42.75“W). (A) Distribution of the Caatinga biome in Brazil and South America. (B) Sampling site within the Caatinga. (C) Placement of mesh seed bags on the soil surface prior to burial.

Fully mature fruits of two annual herbaceous species (*Centrosema pascuorum* Mart. ex Benth. and *Crotalaria retusa* L.) and three tree species [*Mimosa ophthalmocentra* Mart. ex Benth., *Mimosa tenuiflora* (Willd.) Poir., and *Piptadenia retusa* (Jacq.) P.G. Ribeiro, Seigler & Ebinger], were collected from a typical Caatinga area in the municipality of São João do Cariri, Paraíba, Brazil (Figure 1). According to the Köppen–Geiger classification, the region has a hot semiarid climate (BSwh), with annual precipitation of 300–750 mm and high evapotranspiration rates (approximately 0.65 mm day^−1^) (Alvares et al., 2013; Moro et al., 2016). Fruits were collected from at least 20 individuals of each species during their natural dispersal period (June to August), then processed in the laboratory to extract the seeds. All empty or visibly damaged seeds were discarded.

### Field experiment: in situ seed longevity and PY release

Freshly extracted seeds were first assessed for the presence of PY by evaluating germination under two temperature regimes based on preliminary tests: 25°C, considered optimal for germination, and 35°C, representing the upper thermal limit at which germination in most species begins to decline. For each species, five replicates of 20 seeds were placed in germination boxes lined with two layers of moistened filter paper and provided with a 12 h photoperiod (white light, 40 μmol m^−2^ s^−1^) and monitored daily for germination until the response stabilized, defined as a period of five consecutive days with no further germination. Seeds were considered nondormant when imbibition occurred during the germination test. Imbibition of nongerminated seeds was confirmed by visible swelling and softening of the seed coat, determined by applying gentle pressure with forceps. Seeds maintained at 25°C that failed to imbibe were mechanically scarified, returned to the same experimental conditions, and monitored again for germination. Because seed‐coat impermeability is the only factor preventing germination in these species, the total germination percentage at 25°C (nonscarified and scarified seeds combined) was used as a criterion to indicate seed viability (for the freshly collected and the exhumed seeds throughout the field experiment).

**Figure 2:**
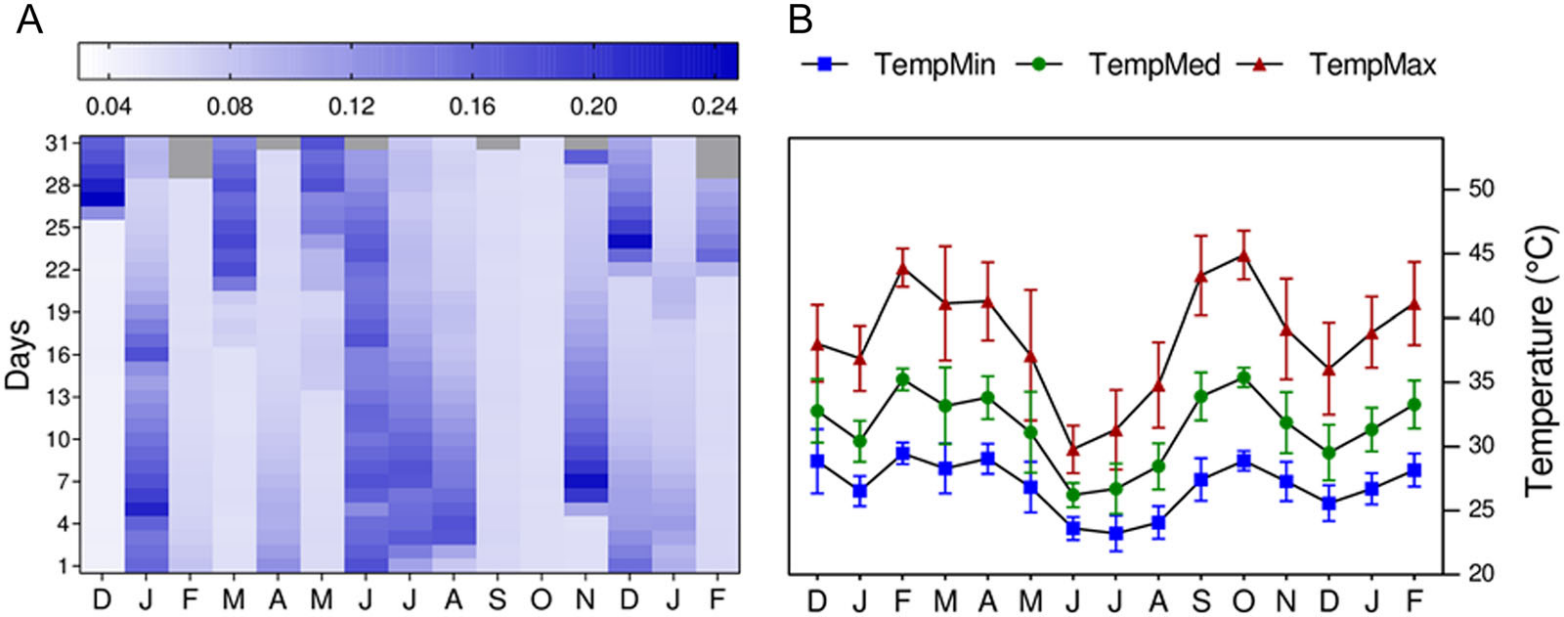
(A) Soil moisture (m^3^ m^–3^) and minimum (min), mean (mean), and maximum (max) soil temperatures (B) recorded during the 15‐month seed burial for five Fabaceae species in the Caatinga in the municipality of São João do Cariri, Paraíba, Brazil.

For the field experiment, freshly collected seeds of each species were divided into 50 replicates of 100 seeds, placed in nylon mesh bags, and buried at a depth of 5 cm at the seed collection site (Matheus et al., 2017; Rodrigues‐Junior et al., 2018). Five bags per species were exhumed after 3, 6, 12, and 15 months, and the number of remaining seeds per bag was recorded at each sampling interval. From each of the five exhumed bags at each time interval, 20 seeds that had no visible physical damage were tested at 25°C and 35°C for PY and germinability as described above the same for the freshly collected seeds. Seeds that failed to imbibe at 25°C were mechanically scarified and monitored again for germination. Soil moisture and temperature were continuously monitored and recorded during the experiment using 5TM Moisture/Temp sensors (METER Group, Pullman, WA, USA) connected to an EM50 datalogger (METER Group). The sensors were buried adjacent to the seed bags, and the recorded soil moisture and temperature data are presented in Figure 2.

### Controlled laboratory experiment: effects of temperature and moisture on PY release

To investigate the effects of temperature and moisture on PY break, we stored freshly collected, non‐scarified seeds of five Fabaceae species under varying temperature and moisture conditions for 6 months. The temperature treatments were the same as those used in the germination tests of freshly harvested and exhumed seeds (25°C and 35°C). In addition, to examine the effects of temperature fluctuations on PY break, an alternating temperature regime of 25°/35°C (12 h/12 h) was included.

To examine the effects of moisture, seeds from all five species were subjected to one of four moisture regimes at each temperature. (A) Dry storage. Seeds were kept in sealed germination boxes to prevent moisture ingress. After 2, 4 and 6 months of dry storage, four replicates of 25 seeds were placed on two layers of filter paper moistened with distilled water, at the same temperature as those in which they had been stored, and the proportion of imbibed (nondormant) seeds was recorded. (B) Wet–dry cycles. To simulate the hydration and dehydration cycles during the rainy season in the Caatinga, where rainfall events are highly irregular and soil moisture pulses may persist for varying periods, we placed seeds in germination boxes and subjected them to alternating periods of moisture and dryness. During the wet phase, water was placed in the boxes for a determined period, then the seeds transferred to dry boxes for a dry period of the same duration. To simulate soil moisture windows of different durations, we exposed seeds either (1) six short cycles (SC) of 2 weeks of moisture, then 2 weeks of no water (dry) or (2) three long cycles (LC) of 4 weeks of moisture, then 4 dry weeks. For both SC and LC treatments, the proportion of seeds that had imbibed water was recorded at the end of each wet period. As experimental controls (C), we used the percentage of freshly collected seeds that had imbibed water after 5 days of exposure to each respective temperature.

### Statistical analyses

R version 4.3.2 (R Core Team, 2023) was used for all analyses. Differences in final germination percentages across treatments were assessed using generalized linear models (GLMs). To evaluate changes in the germination of intact exhumed seeds during the field experiment, we applied a GLM with a quasibinomial distribution and a logit link function with species, temperature, time, and their interactions as predictors. To assess changes in the proportion of remaining and viable seeds, we used two additional GLMs—one with a binomial and the other with a quasibinomial distribution—both using a logit link and including species, time, and their interactions as explanatory variables.

For the controlled conditions experiment, a GLM with a quasibinomial distribution and logit link was used to test the effects of species, moisture regime (dry, short cycles, long cycles), temperature (25°, 25°/35°, and 35°C), storage duration (0, 2, 4, or 6 months), and their interactions on the final proportion of imbibed seeds at the end of the experiment. Quasibinomial error structures were used where appropriate to account for overdispersion detected during model checking with functions from the R package DHARMa (Hartig, 2025).

Predictor significance was assessed using likelihood ratio tests in the R package car (Fox and Weisberg, 2018), followed by Sidak‐adjusted contrasts using the R package emmeans (Lenth et al., 2024). In some field treatments, all five replicates had either 0% or 100% germination, precluding standard post hoc tests, so two of those replicates were adjusted to 1% or 99%, respectively, and the analyses were rerun.

## RESULTS

**Figure 3:**
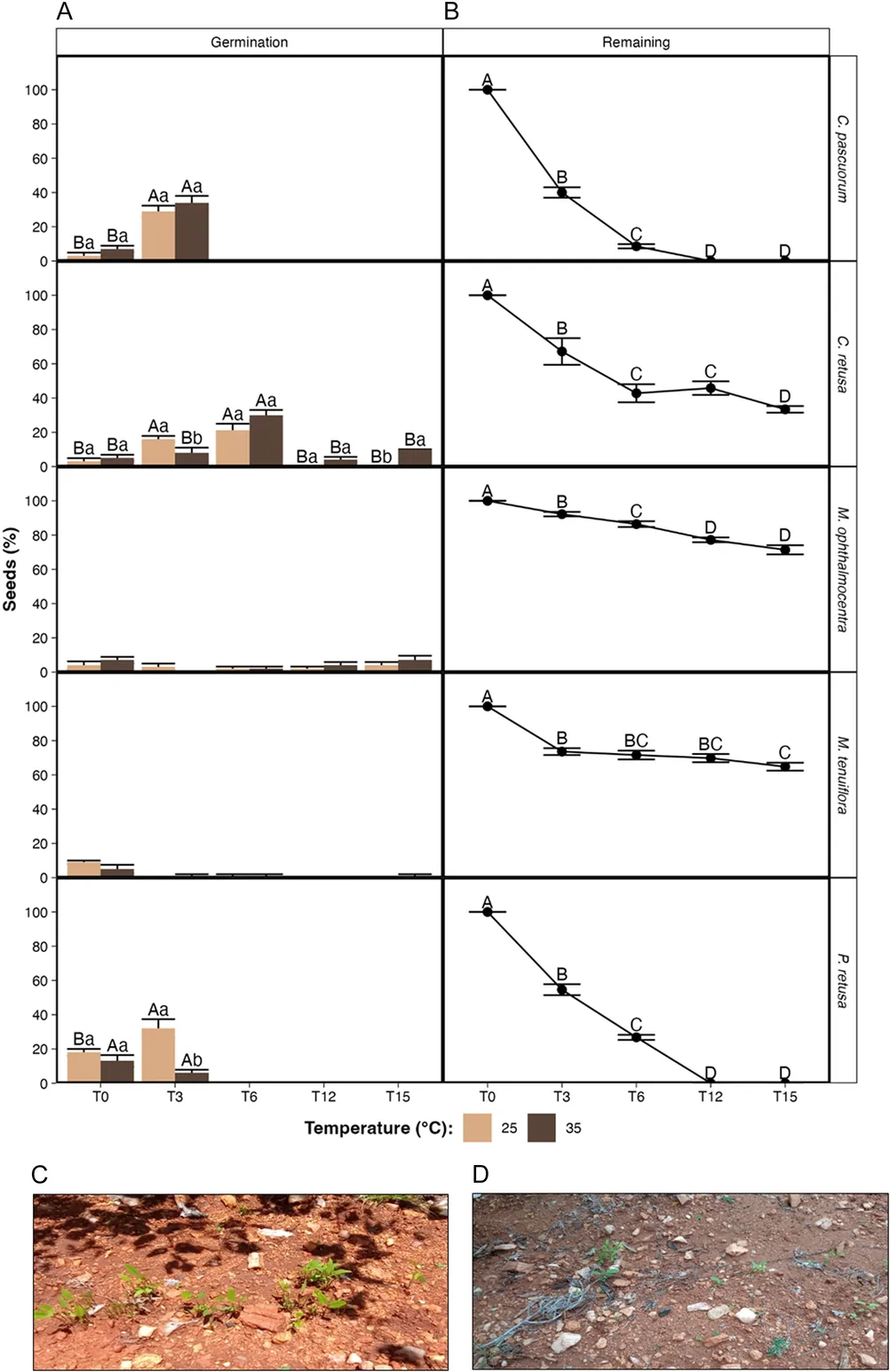
Germination and dormancy of seeds from five Fabaceae species native to the Caatinga, during a 15‐month burial in mesh bags in soil. (A) Germination percentage of nonscarified seeds collected at time zero (T0) and exhumed after 3 (T3), 6 (T6), 12 (T12), and 15 (T15) months. Uppercase letters indicate a significant difference in germination for a species between time points within the same temperature; lowercase letters indicate a difference between temperatures within each time point. (B) Decline in the percentage of seeds remaining in mesh bags in the soil over time. Uppercase letters indicate a significant difference in percentage among time points for a species. (C) Seedling emergence of *C. pascuorum* and (D) *C. retusa* from buried bags after the first rainfall events.

The studied species had distinct responses in terms of PY release, which consequently affects their potential to form persistent soil seed banks (Figure 3A; Appendix S1). The germination percentage of exhumed seeds was not significantly affected by incubation temperature (*χ* ^2^ = 0.033, df = 1, *P* = 0.856; Appendix S2) but varied significantly across burial times (*χ* ^2^ = 72.319, df = 4, *P* < 0.001; Figure 3A) and among species (*χ* ^2^ = 234.637, df = 4, *P* < 0.001). The number of seeds in the exhumed bags decreased over time for all species, although the rate of decline differed among them (Appendix S1).

After 3 months of burial, approximately 40% of *C. pascuorum* seeds germinated without scarification, in contrast to less than 10% of the freshly collected seeds (Figure 3A). Total germination (i.e., the sum of germination of nonscarified and scarified seeds) after burial was similar to that of freshly collected seeds (Table 1), indicating that seed viability was maintained during this period. A marked reduction in the number of seeds in the bags was observed for *C. pascuorum* after 3 months, coinciding with the abundant emergence of seedlings originating from buried seeds in the field (Figure 3B, C). Consequently, after 6 months, mainly due to field germination, fewer than 10% of the original seeds remained in the bags, preventing further germination tests in the laboratory. For *C. retusa*, the germination percentage of nonscarified seeds increased from approximately 5% (freshly harvested) to around 30% after 6 months of burial. The proportion of *C. retusa* seeds remaining in the bags declined to 40% by the sixth month and remained stable at that level until the end of the experiment (Figure 3D). Seeds of this species maintained a high germinative capacity throughout the burial period, such that seeds exhumed after 15 months, when scarified, exhibited a germination percentage of 80% (Table 1).

**Table 1:** Mean total germination (%) of seeds from five Fabaceae species at time of collection (time 0) and after 3, 6, 12, and 15 months of burial in the soil. Total germination (nonscarified + scarified seeds) was used as a proxy for seed viability, with scarification applied to non‐imbibed seeds to overcome physical dormancy (PY). Different letters after values for a species indicate a significant difference in germination percentage between burial duration.

Time buried (months)					
	*C. pascuorum*	*C. retusa*	*C. pascuorum*	*M. tenuiflora*	*C. pascuorum*
0	98 ± 2.00 A	96 ± 1.87 A	93 ± 2.55 A	95 ± 1.58 A	88 ± 3.74 A
3	98 ± 2.00 A	88 ± 2.00 A	94 ± 1.87 A	98 ± 2.00 A	34 ± 6.00 B
6	–	90 ± 5.48 A	94 ± 2.45 A	72 ± 5.83 B	3 ± 2.00 C
12	–	100 ± 0.00 A	98 ± 2.00 A	90 ± 4.47 AB	–
15	–	80 ± 4.47 A	92 ± 3.74 A	96 ± 2.45 A	–

The proportion of seeds with PY observed in freshly collected samples of the tree species *M. ophthalmocentra* and *M. tenuiflora* (~95%) remained throughout the field experiment (Figure 3B). For both species, the number of seeds in the bags decreased slightly after burial for 3 and 6 months and then stabilized, with approximately 70% of the seeds still present after 15 months (Figure 3B). For *M. ophthalmocentra* and *M. tenuiflora*, the total percentage of germinated seeds (non‐scarified + scarified) after 15 months was 92% and 96%, respectively (Table 1). In contrast, all exhumed seeds of *P. retusa* had imbibed without scarification after the 3‐month burial (Figure 3B); however, only 34% of these seeds germinated. None of the seeds of *P. retusa*, exhumed after 6 months in the soil seed bank were viable (Table 1).

**Figure 4:**
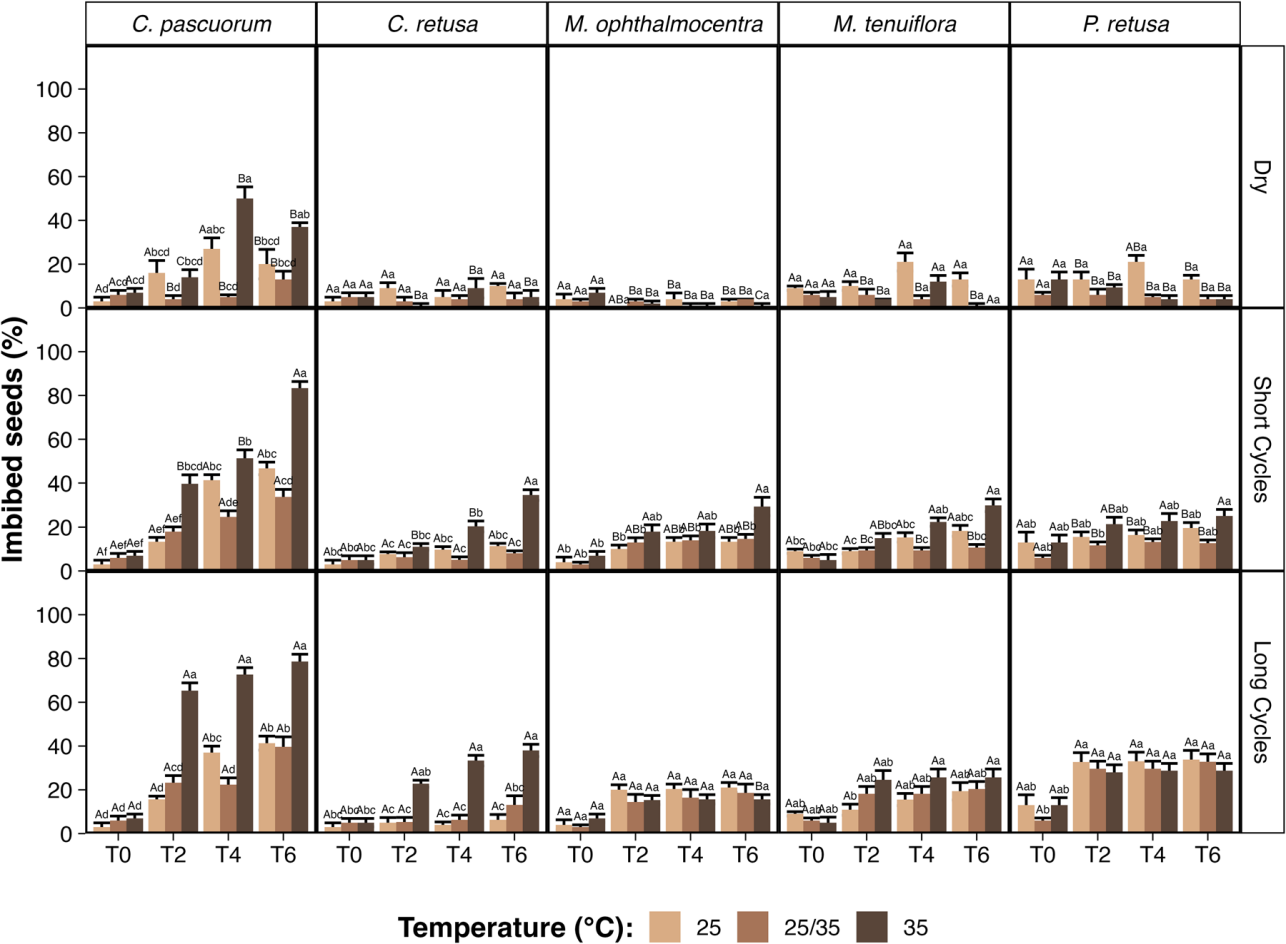
Percentage of imbibed (dormancy release) seeds of five Fabaceae species from the Caatinga exposed to three different temperature and moisture regimes for 6 months. Uppercase letters indicate a significant difference in percentages among species at the same time and temperature; lowercase letters a significant difference between different temperatures and times for a moisture treatment.

In the laboratory experiment, temperature (*χ* ^2^ = 527.21, df = 2, *P* < 0.001) and moisture (*χ* ^2^ = 463.04, df = 2, *P* < 0.001) significantly influenced PY release (Figure 4). These factors interacted significantly with time and species (Appendix S3). The relative importance of temperature and moisture varied among species. However, the highest levels of dormancy release were consistently observed in seeds exposed to moisture conditions, regardless of cycle duration (short or long). With respect to temperature, the 25°C and 25°/35°C treatments had comparatively weaker effects on PY release than constant 35°C (Appendix S3).

The herbaceous species *C. pascuorum* had the highest proportions of nondormant seeds in all storage conditions (Figure 4). For seeds exposed to the wet–dry cycles (SC and LC), the proportion of nondormant seeds of *C. pascuorum* stored at 35°C was 40% higher than that of nondormant seeds stored in the dry conditions (Figure 4). Although germination increased significantly in seeds exposed to wet–dry cycles at 25°C and 25°/35°C, the proportion of nondormant *C. pascuorum* seeds remained below 50% (Figure 4). The proportion of nondormant seeds of *C. retusa* after 6 months at 35°C in the short wet–dry cycles and the long was 40% (Figure 4). No change in dormancy status was observed for this species in the other treatments.

In the controlled conditions, the tree species *M. ophthalmocentra*, *M. tenuiflora*, and *P. retusa* had lower percentages of nondormant seeds among the total seeds compared to the herbaceous species *C. pascuorum* and *C. retusa* (Figure 4). No significant change in dormancy was detected for any of these species in dry storage. However, under the alternating wet–dry cycles, regardless of the temperature regime, the proportion of non‐dormant seeds at the end of the experiment was 20–30% higher than at the beginning (Figure 4). In *M. ophthalmocentra* and *M. tenuiflora*, the highest levels of PY release occurred in seeds stored at 35°C under moist conditions, particularly in SC treatment, which resulted in the greatest proportion of non‐dormant seeds (Figure 4).

## DISCUSSION

The release of physical dormancy (PY) in Fabaceae seeds from the Caatinga, and thus their potential to form persistent soil seed banks, was mediated by interactions between temperature and moisture and varied among the studied species, with herbaceous species losing dormancy more rapidly than tree species. Several studies have emphasized the synergistic role of temperature and moisture in promoting PY break in Fabaceae seeds across diverse ecosystems (van Klinken and Goulier, 2013; Fidelis et al., 2016; Matheus et al., 2017; Rodrigues‐Junior et al., 2018; Zhang et al., 2020; Just et al., 2024). Seeds exhibiting PY have a water‐impermeable seed coat with anatomical regions that respond to fracture in specific conditions (Jayasuriya et al., 2015; Rodrigues‐Junior et al., 2018; Jaganathan and Harrison, 2024). Here we demonstrated that exposure to alternating wet–dry cycles and elevated temperatures—environmental conditions characteristic of semiarid ecosystems such as the Caatinga (Meiado et al., 2012; Gomes et al., 2023)—promoted PY break and subsequent germination. These findings indicate that PY influences seed longevity in the soil seed bank and that Caatinga climatic conditions promote a gradual release of dormancy, dispersing germination over time in a manner dependent on species growth form, thereby supporting our hypothesis.

The Fabaceae species had distinct responses regarding their potential to form persistent soil seed banks. For the herbs *C. pascuorum* and *C. retusa*, the number of seeds in the buried bags markedly declined shortly after the onset of the first rains, coinciding with substantial seedling emergence in the field. The reduction in seed number in the buried bags is consistent with findings for other Fabaceae species (Marques et al., 2014; Matheus et al., 2017). In contrast, seeds of the tree species maintained high levels of dormancy and viability over time, supporting a persistence strategy based on delayed germination. For these species, PY represents an adaptive strategy that enhances seedling survival by spreading germination events across time (Jaganathan, 2016; Kiss et al., 2018; Jaganathan and Harrison, 2024). Variation in PY release among species may be associated with differences in physical dormancy intensity, defined as the degree of resistance of the seed coat to environmental conditions that promote dormancy break (Baskin et al., 1998). Species with lower dormancy intensity tend to lose PY more rapidly when exposed to favorable temperature and moisture conditions, whereas species with higher dormancy intensity maintain impermeable seed coats for longer periods, delaying dormancy release and increasing the likelihood of persistence in the soil. Such variation has been reported for several species across distinct ecosystems (Miller et al., 2019; Huss and Gierlinger, 2021; Moro et al., 2024).

In *P. retusa*, the rapid loss of viability following burial, despite early dormancy release, indicates a decoupling between PY and seed longevity, in contrast with patterns observed for other Fabaceae (Long et al., 2015; Moro et al., 2021, 2024) and suggests that, in this species, persistence in the soil is constrained primarily by mortality rather than by dormancy dynamics. The decline observed for *P. retusa* was mainly due to seed mortality, similar to that reported for *Dimorphandra wilsonii*, another tropical Fabaceae species (Matheus et al., 2017), instead of primarily to germination events as found for buried seeds of the herbaceous species. Dry‐stored seeds of *P. retusa* also gradually declined in dormancy and viability over 4 years (H. A. Almeida, personal observation). These results support the assertion of Thompson et al. (2003) that PY is not a universal predictor of seed longevity.

Rainfall‐driven increases in soil moisture during the first 3 months of burial promoted seedling emergence from the burial bags, particularly for *C. pascuorum*, indicating rapid alleviation of PY in the field. This pattern was accompanied by a marked decline in the number of seeds inside the buried bags, consistent with the formation of a transient soil seed bank (<12 months, sensu Thompson, 1993). In contrast, although seedlings of *C. retusa* also emerged, a substantial fraction of seeds remained in the soil with high viability by the end of the experiment, supporting its classification as forming a short‐term persistent seed bank (≥12 months). Of the three tree species, *M. ophthalmocentra* and *M. tenuiflora* maintained high seed survival and viability over the burial period, indicating strong potential to form persistent soil seed banks. Conversely, the rapid decline in viability observed in *P. retusa*, coupled with limited seedling emergence, reinforces that its seed persistence is constrained primarily by mortality rather than germination. Together, these patterns reveal pronounced interspecific variation in the balance between dormancy release, germination, and seed survival, which ultimately determines soil seed bank persistence in these Fabaceae species.

Although previous studies have highlighted the positive influence of temperature fluctuations on the alleviation of PY (Jaganathan, 2018; Jaganathan and Harrison, 2024), this relationship was not observed for the Caatinga species investigated in this study. These findings are consistent with those reported by van Klinken and Goulier (2013) for other Fabaceae species, in which alternating temperature regimes did not significantly enhance the breakdown of PY. *Centrosema pascuorum* and *C. retusa* exhibited a more pronounced dormancy release response when exposed to wet–dry cycles at 35°C. In *C. retusa*, temperature exerted a stronger effect than moisture (SC or LC), a result also observed in *M. ophthalmocentra* and *M. tenuiflora*, albeit to a lesser extent. The sensor data showed that, between pulses of soil moisture (Figure 2A), seeds were exposed to temperatures exceeding 45°C (Figure 2B). In this regard, consistent with previous findings for the tropical species *D. wilsonii* (Matheus et al., 2017) and *Senna multijuga* (Rodrigues‐Junior et al., 2018a), the interaction between temperature and moisture likewise acts as a key driver of PY release in Fabaceae species from the Caatinga.

This study showed that seeds of the annual herbs *C. pascuorum* and *C. retusa* lost dormancy more rapidly than the tree species *M. ophthalmocentra* and *M. tenuiflora*. According to Baskin et al. (1998), seeds of tree species tend to exhibit more intense PY than those of annual herbs, which may lead to variation in the temporal dynamics of dormancy release among the different species composing the seed bank. A study of seed bank composition in the Caatinga over time found that annual herbs tend to form transient seed banks, while perennial species typically form persistent ones (Souza et al., 2020). Annual herbs in the Caatinga generally develop rapidly and complete their life cycles within the short windows of soil moisture provided by the region's brief and irregular rainfall events (Albuquerque et al., 2012; Santos et al., 2013). These species adjust the duration of their phenophases in response to rainfall intensity and duration (Aguiar et al., 2024). Thus, for annual herbs, lower PY intensity and its timely release at the start of the rainy season may enhance seedling establishment compared to perennial species. In contrast, tree species require longer periods to reach developmental stages capable of withstanding prolonged drought in the Caatinga (Meiado et al., 2012; Ribeiro et al., 2021). As a result, seeds typically germinate at different times, improving long‐term recruitment potential. These contrasting germination strategies reflect distinct adaptive responses to seasonal constraints in the Caatinga, highlighting the key role of PY in regulating seedling recruitment and maintaining the functional diversity of species in this ecosystem. These findings contribute to our understanding of the reproductive strategies of Caatinga Fabaceae and offer valuable insights for conservation and management efforts within this biome and globally for other seasonally dry tropical forests.

## AUTHOR CONTRIBUTIONS

H.A.A.: conceptualization; data curation; formal analysis; investigation; methodology; writing original draft. J.A.S.S.: conceptualization; data curation; methodology; review and editing. Q.S.G.: conceptualization; methodology; supervision; validation; review and editing.

## CONFLICT OF INTEREST STATEMENT

The authors declare no conflicts of interest.

## Supporting information


**Appendix S1:** Predictor significance for the model on the proportion of remaining and viable seeds from the field experiment, based on the likelihood ratio test.

**Appendix S2:** Analysis of variance from the general effects model for the percentage of imbibed seeds, considering the main effects and interactions between temperature and burial duration.

**Appendix S3:** Predictor significance for the laboratory experiment based on the likelihood ratio test.

## Data Availability

Data associated with this study have been deposited at Figshare (https://doi.org/10.6084/m9.figshare.33383902).
