# Supplementary material for: Humidity and temperature interact in breaking physical dormancy in Fabaceae seeds from the Brazilian semiarid region
Source: Am J Bot. 2026 Sep 4;113(9):e70254. doi: 10.1002/ajb2.70254 (PMC13595477; doi:10.1002/ajb2.70254)
Supplement: Supplementary file 1 [file AJB2-113-e70254-s001.docx]

**Appendix S1.** Predictor significance for the model on the proportion of remaining and viable seeds from the field burial experiment based on the likelihood ratio test.

|  | **Remaining seeds** | | | **Viable seeds** | | |
| --- | --- | --- | --- | --- | --- | --- |
| **Predictor** | ***χ*²** | **df** | ***P*** | ***χ*²** | **df** | ***P*** |
| Time | 4809.7 | 4 | <0.001 | 259.3 | 4 | <0.001 |
| Species | 3470.8 | 4 | <0.001 | 107.3 | 4 | <0.001 |
| Time:Species | 624.0 | 16 | <0.001 | 74.4 | 11 | <0.001 |
