# Supplementary material for: Humidity and temperature interact in breaking physical dormancy in Fabaceae seeds from the Brazilian semiarid region
Source: Am J Bot. 2026 Sep 4;113(9):e70254. doi: 10.1002/ajb2.70254 (PMC13595477; doi:10.1002/ajb2.70254)
Supplement: Supplementary file 2 [file AJB2-113-e70254-s002.docx]

**Appendix S2.** Analysis of variance from the general effects model for the percentage of imbibed seeds, considering main effects and interactions between temperature (25°C and 35°C) and burial duration (3, 6, 12, and 15 months).

| **Predictor** | ***χ*²** | **df** | ***P*** |
| --- | --- | --- | --- |
| Temperature | 0.033 | 1 | 0.856 |
| Time | 72.319 | 4 | <0.001 |
| Species | 234.637 | 4 | <0.001 |
| Temperature:Time | 38.642 | 4 | <0.001 |
| Temperature:Species | 35.485 | 4 | <0.001 |
| Time:Species | 171.210 | 11 | <0.001 |
| Temperature:Time:Species | 17.649 | 12 | 0.127 |
