# Supplementary material for: Humidity and temperature interact in breaking physical dormancy in Fabaceae seeds from the Brazilian semiarid region
Source: Am J Bot. 2026 Sep 4;113(9):e70254. doi: 10.1002/ajb2.70254 (PMC13595477; doi:10.1002/ajb2.70254)
Supplement: Supplementary file 3 [file AJB2-113-e70254-s003.docx]

**Appendix S3.** Predictor significance for the laboratory experiment based on the likelihood ratio test.

| **Predictor** | ***χ*²** | **df** | ***P*** |
| --- | --- | --- | --- |
| Humidity | 464.04 | 2 | <0.001 |
| Temperature | 527.21 | 2 | <0.001 |
| Time | 610.06 | 3 | <0.001 |
| Species | 1210.03 | 4 | <0.001 |
| Humidity:Temperature | 66.19 | 4 | <0.001 |
| Humidity:Time | 86.95 | 6 | <0.001 |
| Temperature:Time | 21.16 | 6 | 0.002 |
| Humidity:Species | 57.99 | 8 | <0.001 |
| Temperature:Species | 307.90 | 8 | <0.001 |
| Time:Species | 180.04 | 12 | <0.001 |
| Humidity:Temperature:Time | 44.63 | 12 | <0.001 |
| Humidity:Temperature:Species | 95.64 | 16 | <0.001 |
| Humidity:Time:Species | 17.98 | 24 | 0.804 |
| Temperature:Time:Species | 61.09 | 24 | <0.001 |
| Humidity:Temperature:Time:Species | 43.30 | 48 | 0.665 |
